# Effect of non-lattice oxygen on ZrO_2_-based resistive switching memory

**DOI:** 10.1186/1556-276X-7-187

**Published:** 2012-03-14

**Authors:** Chun-Chieh Lin, Yi-Peng Chang, Huei-Bo Lin, Chu-Hsuan Lin

**Affiliations:** 1 Department of Electrical Engineering, National Dong Hwa University, Hualien, 97401, Taiwan; 2 Department of Opto-Electronic Engineering, National Dong Hwa University, Hualien, 97401, Taiwan

**Keywords:** ZrO_2_, resistive switching memory, non-lattice oxygen, retention time, resistive switching mechanism

## Abstract

ZrO_2_-based resistive switching memory has attracted much attention according to its possible application in the next-generation nonvolatile memory. The Al/ZrO_2_/Pt resistive switching memory with bipolar resistive switching behavior is revealed in this work. The thickness of the ZrO_2 _film is only 20 nm. The device yield improved by the non-lattice oxygen existing in the ZrO_2 _film deposited at room temperature is firstly proposed. The stable resistive switching behavior and the long retention time with a large current ratio are also observed. Furthermore, it is demonstrated that the resistive switching mechanism agrees with the formation and rupture of a conductive filament in the ZrO_2 _film. In addition, the Al/ZrO_2_/Pt resistive switching memory is also possible for application in flexible electronic equipment because it can be fully fabricated at room temperature.

## Introduction

Lately, a novel memory device, resistive switching memory, has been extensively studied due to its great potential of low operation voltage, low power consumption, high operation speed, nonvolatility, and simple structure [1-8]. Particularly, the ZrO_2_-based resistive switching memory has attracted more and more attention because it is compatible with the conventional CMOS process [1,2]. In the previous reports [9-13], the Al/ZrO_2_/Pt structural devices presented a unipolar resistive switching property that might cause a switching error while the unipolar resistive switching was performed. However, the Al/ZrO_2_/Pt device with bipolar resistive switching is revealed in this work. It is demonstrated that the device with bipolar resistive switching is more stable and reliable for memory application. In addition, the existence of non-lattice oxygen in the ZrO_2 _film deposited at room temperature (RT) is firstly proposed. We infer that the non-lattice oxygen will react with the Al atoms to form an AlO_y _interface layer during the deposition of the Al top electrode (TE). The resistive switching within the interface layer is expected to be more stable and uniform than that within the bulk ZrO_2 _film, leading to a higher device yield [9]. The Al/ZrO_2_/Pt device proposed in this study is also possible for application in flexible electronic equipment because it can be fully fabricated at RT.

## Experimental details

**Figure 1:**
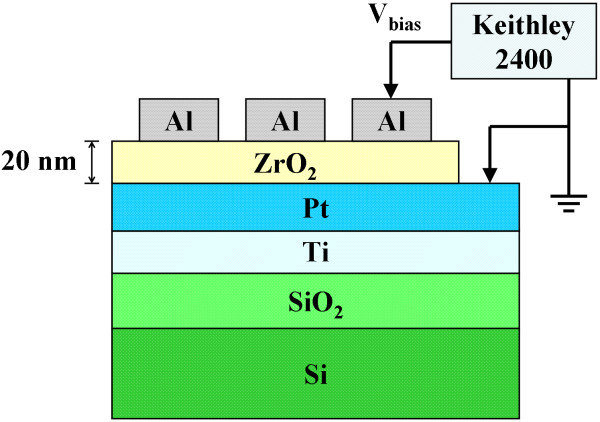
**Structure of the Al/ZrO_2_/Pt device**. Throughout the electrical measurements, bias voltages were applied on the Al TE; meanwhile, the Pt BE was grounded.

As shown in Figure 1, the device that consisted of five sets of samples in the form of Al/ZrO_2_/Pt sandwich structure was employed in this work. First of all, a 200-nm-thick SiO_2 _isolation layer was thermally grown on five cleaned Si substrates in an oxidation furnace. After that, a 20-nm-thick Ti adhesion layer and a 60-nm-thick Pt bottom electrode (BE) were continuously deposited on the SiO_2 _layer by an electron beam evaporator at RT without breaking a vacuum. Then, an about 20-nm-thick ZrO_2 _resistive switching layer was deposited on the Pt BE by a radio frequency magnetron sputtering at different temperatures, including RT, 150°C, 200°C, 250°C, and 300°C. Finally, 300-nm-thick Al TEs with 250-μm diameter defined by a shadow mask were deposited on the ZrO_2 _film at RT by the sputtering to complete the Al/ZrO_2_/Pt structural samples.

The chemical bonding states and the non-lattice oxygen of the ZrO_2 _films were determined by an X-ray photoelectron spectroscopy (XPS). The electrical properties of the samples were recorded by Keithley 2400 source meter (Keithley Instruments, Inc., Cleveland, OH, USA). Throughout the electrical measurements, bias voltages were applied on the Al TE; meanwhile, the Pt BE was grounded. All of the measurements were performed at RT.

## Results and discussion

**Figure 2:**
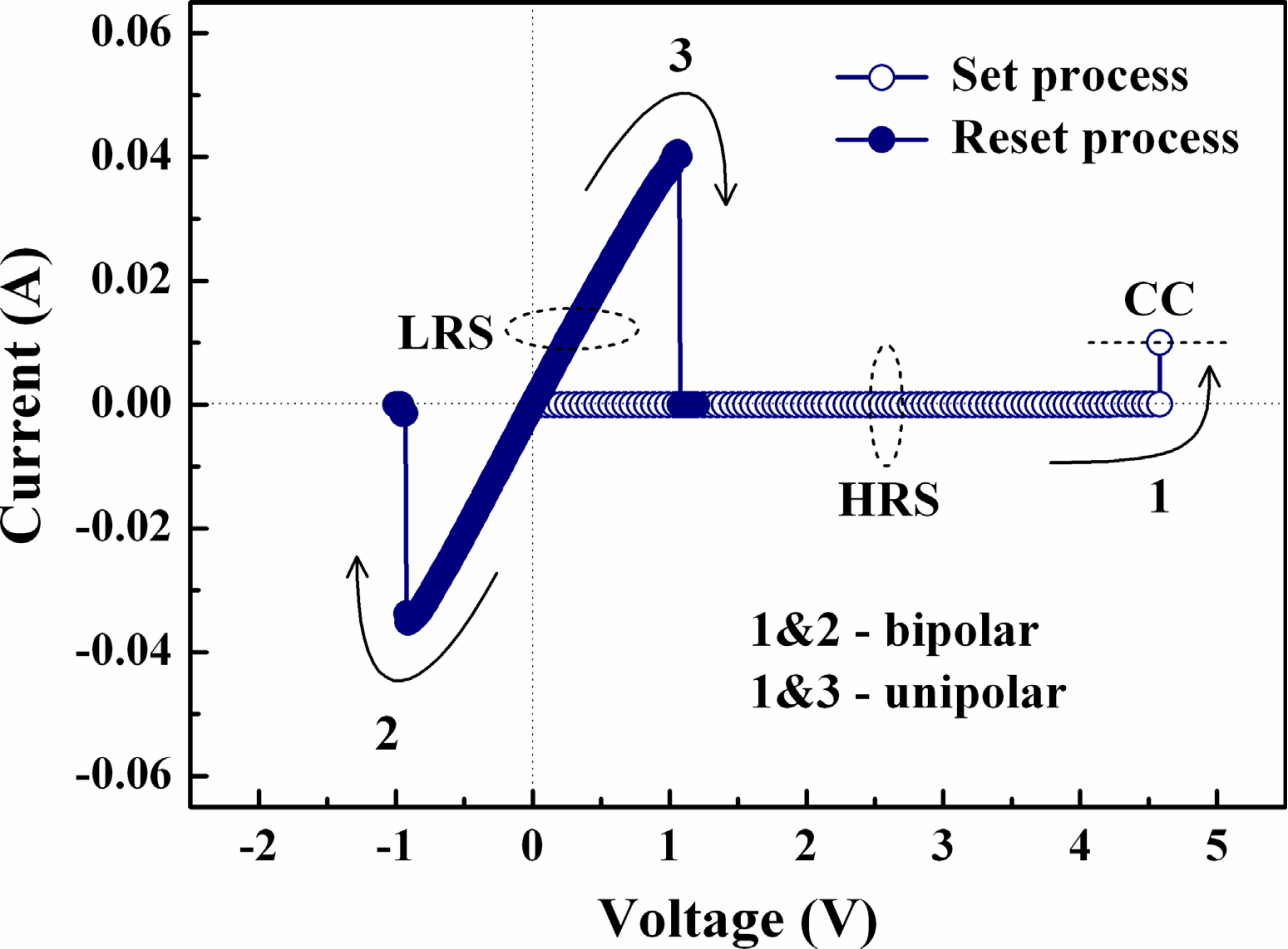
**Typical resistive switching *I*-*V *curves of the Al/ZrO_2_/Pt device**. Under the bipolar resistive switching mode (curves 1 and 2) and the unipolar resistive switching mode (curves 1 and 3).

Figure 2 depicts the resistive switching *I*-*V *curves typically for the Al/ZrO_2_/Pt samples where the ZrO_2 _films were deposited at various temperatures. The bipolar resistive switching indicates that the memory states of the device are altered by applying bias voltages with different polarities (curves 1 and 2). On the other hand, the unipolar resistive switching means that the memory states are switched by applying bias voltages with the same polarity (curves 1 and 3). As shown in Figure 2, the memory states of the device can be switched from a high resistance state (HRS) to a low resistance state (LRS) by applying a positive bias voltage, which is called set process. Besides, the memory states can be altered back to the HRS by applying a positive or negative bias, called reset process. The reset process is independent of voltage polarity. A current compliance (CC) is set at 10 mA during the set process to prevent degradation of the device, but no CC is used during the reset process. The set and reset processes, i.e., the resistive switching, can be stably repeated for a lot of times, and the memory states between the LRS and HRS are distinguishable.

**Figure 3:**
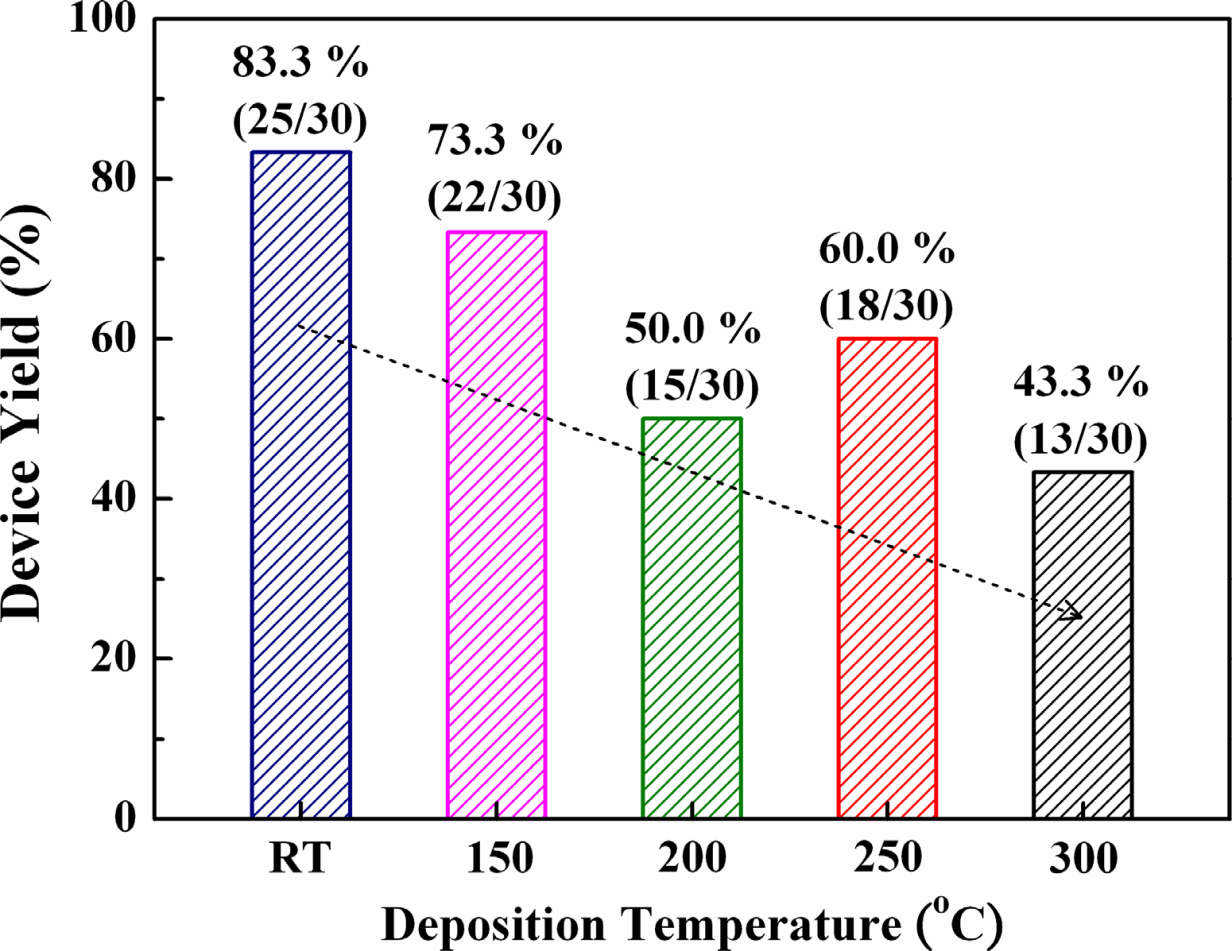
**Yields of the Al/ZrO_2_/Pt samples deposited at various temperatures**. Where the ZrO_2 _films were deposited at RT, 150°C, 200°C, 250°C, and 300°C. The samples fabricated at RT show the highest yield.

**Figure 4:**
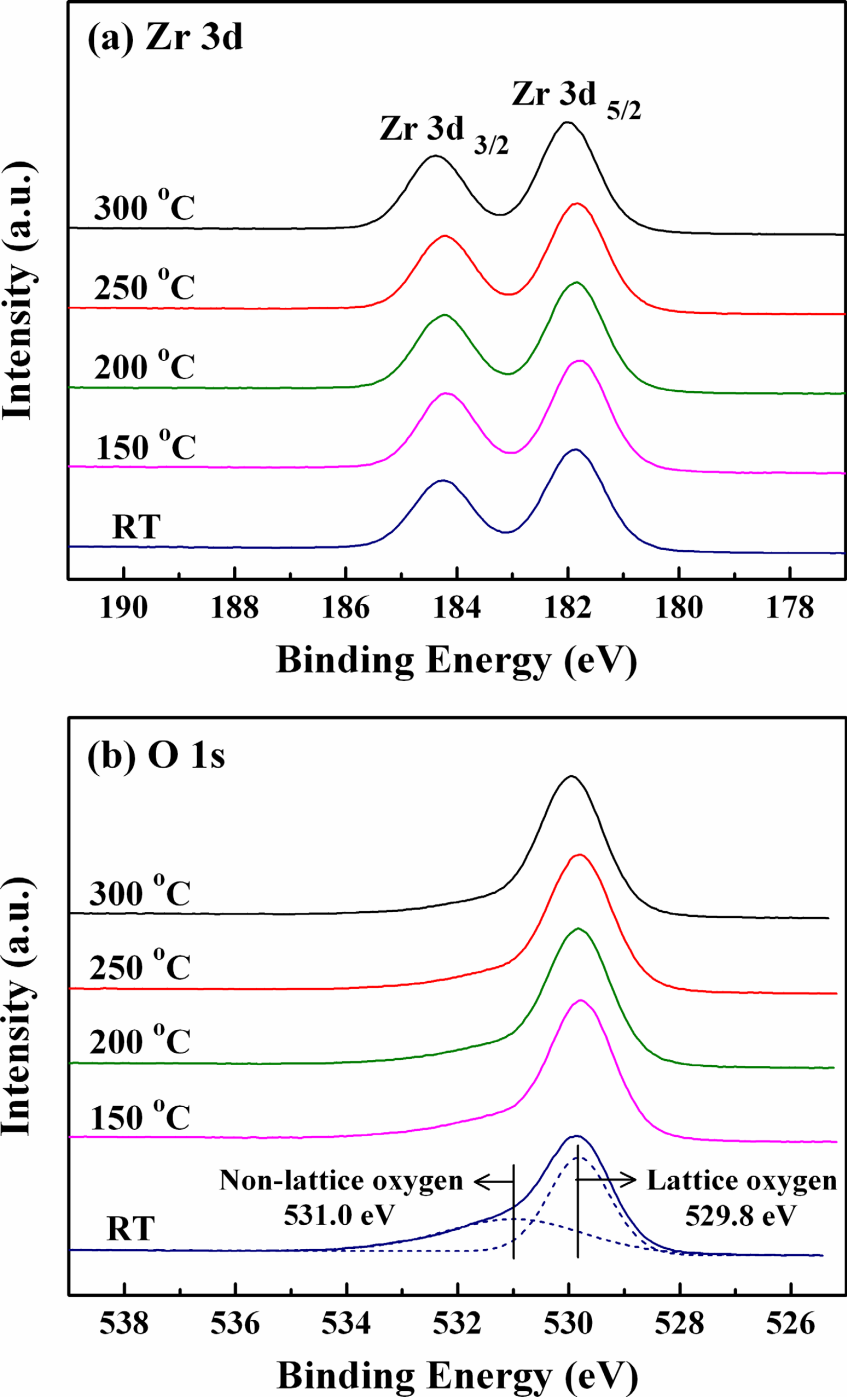
**XPS spectra of the ZrO_2 _films deposited at various temperatures**. (**a**) Zr 3d and (**b**) O 1s XPS spectra of the ZrO_2 _films deposited at RT, 150°C, 200°C, 250°C, and 300°C. The ZrO_2 _film deposited at RT possesses the highest content of the non-lattice oxygen.

Figure 3 shows the yield of the samples where the ZrO_2 _films were deposited at various temperatures. The yield is defined as a percentage of samples which possess the resistive switching behavior. As shown in Figure 3, the Al/ZrO_2_/Pt samples where the ZrO_2 _film was deposited at RT show the highest yield. In addition, the device yields are decreasing with the increased deposition temperatures of the ZrO_2 _films, the property which can be explained by the XPS results as shown in Figure 4.

Figure 4a exhibits the Zr 3d XPS spectra of the ZrO_2 _films deposited at various temperatures. The peaks of Zr 3d_3/2 _and Zr 3d_5/2 _are near 184 and 182 eV, respectively, the energy which indicates the ZrO_2 _bonding [14,15]. In addition, no metallic Zr peak (178.9 eV) is found [16], and the result shows that the ZrO_2 _films deposited at various temperatures are fully oxidized. Figure 4b shows the O 1s XPS spectra of the ZrO_2 _films. The ZrO_2 _films deposited at various temperatures exhibit lattice oxygen signals at about 529.8 eV, which indicates the Zr-O bonding [14]. Besides, non-lattice oxygen signals at 531.0 eV decrease with the increased deposition temperatures of the ZrO_2 _films. Therefore, the ZrO_2 _film deposited at RT possesses the highest content of the non-lattice oxygen, where the ZrO_2 _film plays a role of oxygen storage room. During the sputtering of the Al TEs, we infer that the non-lattice oxygen in the ZrO_2 _film will react with the Al atoms to form an AlO_y _interface layer. The resistive switching within the interface layer is expected to be more stable and uniform than that within the bulk ZrO_2 _film, leading to a higher device yield. Lin et al. also demonstrated that the resistive switching near the Ti/ZrO_2 _interface layer with sufficient oxygen ions possesses stable resistive switching behavior [12]. Because the yield of the Al/ZrO_2_/Pt device fabricated at RT is higher than that of the other samples, more detailed investigations focused on this device are shown as follows.

**Figure 5:**
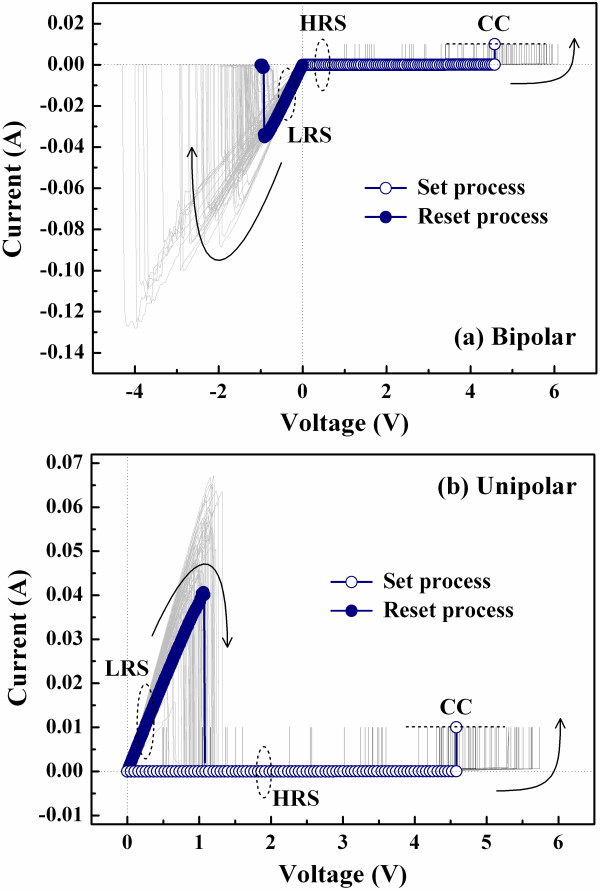
**Resistive switching cycles of the samples fabricated at RT**. Under the (**a**) bipolar and (**b**) unipolar modes. The resistive switching can be stably repeated for over 100 times under both resistive switching modes.

**Figure 6:**
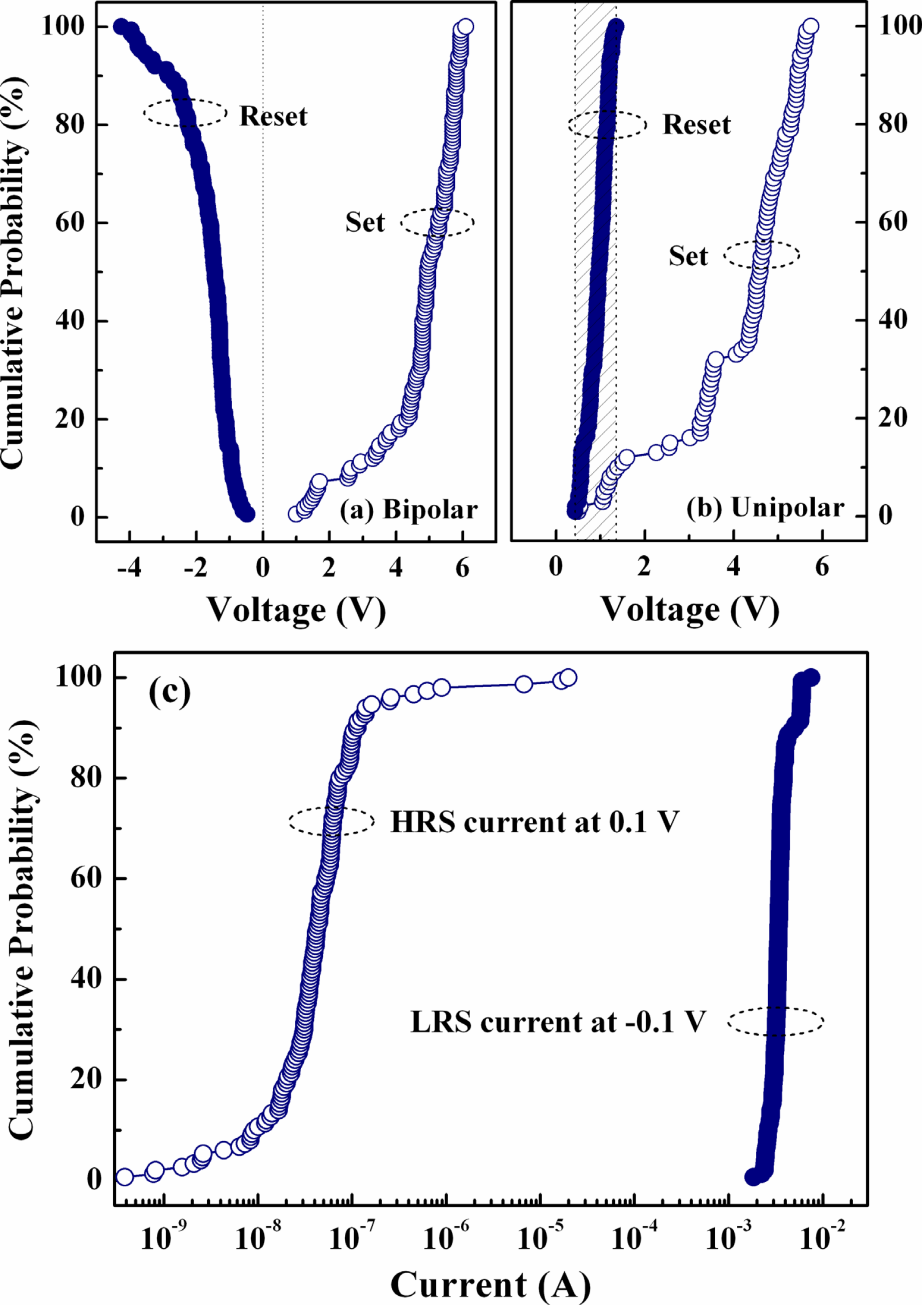
**Cumulative probabilities of the set and reset voltages and the LRS and HRS currents**. Cumulative probabilities of the set and reset voltages of the sample fabricated at RT under the (**a**) bipolar and (**b**) unipolar modes. The set and reset voltages under the bipolar mode are distinguishable. (**c**) Cumulative probabilities of the HRS currents measured at 0.1 V and the LRS currents measured at -0.1 V under the bipolar mode. Two memory states are distinguishable.

Figure 5a, b depicts the resistive switching cycles of the samples fabricated at RT under the bipolar and unipolar resistive switching modes, respectively. The resistive switching can be stably repeated for over 100 times under both resistive switching modes. Figure 6a, b is the cumulative probabilities of the set and reset voltages under the bipolar and unipolar modes depicted in Figures 5a, b, respectively. The set and reset voltages under the bipolar mode are distinguishable; however, the voltages under the unipolar mode show a little overlap as shown in the shaded region of Figure 6b. Figure 6c shows the cumulative probabilities of the HRS currents measured at 0.1 V and the LRS currents measured at -0.1 V under the bipolar mode depicted in Figure 5a. During 150 resistive switching cycles, the LRS currents firmly hold on about several milliampere, and the HRS currents keep very low. Two memory states are distinguishable under the bipolar resistive switching.

**Figure 7:**
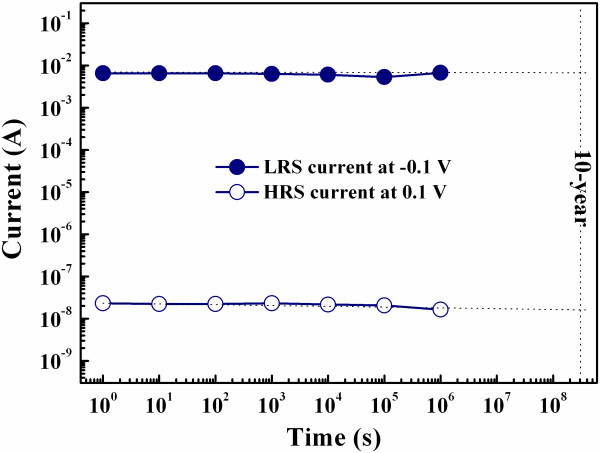
**Retention time of the sample fabricated at RT**. LRS and HRS currents measured at ± 0.1 V firmly hold on about 6 mA and 2 × 10^-8 ^A, respectively, for over 10^6 ^s without applying any power supply, so the good nonvolatility of the sample is demonstrated.

Figure 7 shows the retention time of the sample fabricated at RT under the bipolar resistive switching mode. The LRS and HRS currents measured at ± 0.1 V firmly hold on about 6 mA and 2 × 10^-8 ^A, respectively, for over 10^6 ^s without applying any power supply. The current ratio between the two memory states is over 10^5 ^times. Consequently, the good nonvolatility of the sample is demonstrated.

**Figure 8:**
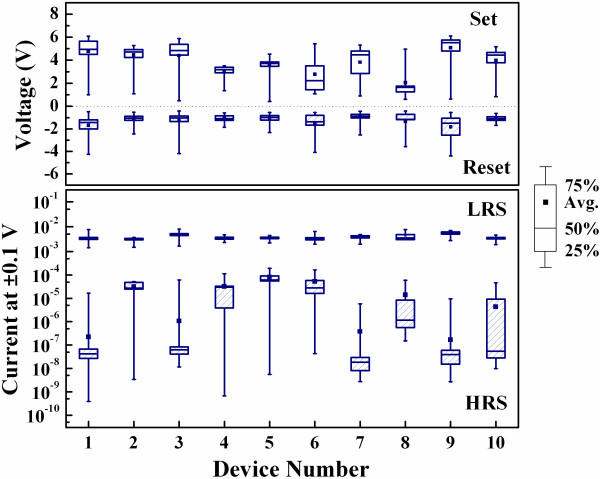
**Device-to-device uniformities of ten samples fabricated at RT under the bipolar resistive switching mode**. Including the set and reset voltages and the LRS and HRS currents measured at ± 0.1 V.

Figure 8 shows device-to-device uniformities of ten samples fabricated at RT under the bipolar resistive switching mode. The set and reset voltages under the bipolar mode are distinguishable. The LRS currents measured at -0.1 V and the HRS currents measured at 0.1 V are also distinguishable.

**Figure 9:**
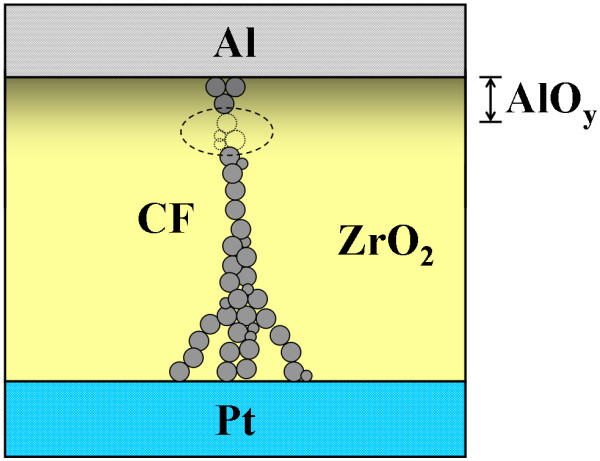
**Possible resistive switching mechanism of the Al/ZrO_2_/Pt device**. The set and reset are due to the formation and rupture of the CF in the ZrO_2 _film.

Based on the resistive switching *I*-*V *curves shown in Figure 2 and the XPS spectra depicted in Figure 4, a possible resistive switching mechanism of the ZrO_2_-based resistive switching memory fabricated at RT is proposed. As shown in Figure 9, we infer that the AlO_y _interface layer with some oxygen vacancies will be formed during the sputtering of the Al TE. While a positive bias voltage is applied on the Al TE, the oxygen vacancies with positive charges will migrate through the ZrO_2 _film to connect the Pt BE, the connection which causes the formation of conductive filament (CF) [17]. On the other hand, the reset process can be achieved by applying a positive or negative bias voltage, i.e., regardless of voltage polarity, so we suppose that the reset process happens due to thermal oxidation of the oxygen vacancies in the CF near the AlO_y_/ZrO_2 _interface by accumulated local Joule heating, causing the rupture of the CF [18,19]. However, migration of charged ions leading to the rupture of the CF can be excluded in this study because the reset process is independent of voltage polarity. In addition, the LRS current of the Al/ZrO_2_/Pt device is dominated by Ohmic conduction, which corresponds to the CF conduction model as shown in Figure 9. Furthermore, the HRS current of the device follows Frenkel-Poole emission at high electric fields, the result which corresponds to the bulk leakage current in the ZrO_2 _film.

## Conclusions

The Al/ZrO_2_/Pt resistive switching memory was successfully fabricated at RT. The thickness of the ZrO_2 _film proposed in this work is only 20 nm. The device yield improved by the non-lattice oxygen in the ZrO_2 _film is demonstrated. The memory states of the device can be set from the HRS to the LRS by applying a positive bias voltage, leading to the migration of oxygen vacancies in the ZrO_2 _film to connect the Pt BE, the connection which causes the formation of the CF. In addition, the memory state can be reset back to the HRS by applying a bias voltage regardless of its polarity, so we suppose that the reset process happens due to thermal oxidation of the oxygen vacancies in the CF by accumulated local Joule heating, causing the rupture of the CF. The nonvolatility of the device is also demonstrated. The Al/ZrO_2_/Pt resistive switching memory is also possible for application in flexible electronic equipment because it can be fully fabricated at RT.

## Abbreviations

BE: bottom electrode; CC: current compliance; CF: conductive filament; HRS: high resistance state; LRS: low resistance state; RT: room temperature; TE: top electrode; XPS: X-ray photoelectron spectroscopy.

## Competing interests

The authors declare that they have no competing interests.

## Authors' contributions

CCL conceived of the study, designed the experiment, and drafted the manuscript. YPC and HBL prepared the devices and carried out the XPS analyses and electrical measurements. CHL participated in the design of the experiment and assisted in the electrical measurements. All authors read and approved the final manuscript.

## References

[B1] Baek IG Lee MS Seo S Lee MJ Seo DH Suh DS Park JC Park SO Kim HS Yoo IK Chung UI Moon JT Highly scalable non-volatile resistive memory using simple binary oxide driven by asymmetric unipolar voltage pulses IEDM Tech Dig 2004 587

[B2] Chen A Haddad S Wu YC Fang TN Lan Z Avanzino S Pangrle S Buynoski M Rathor M Cai W Tripsas N Bill C VanBuskirk M Taguchi M Non-volatile resistive switching for advanced memory applications IEDM Tech Dig 2005 746

[B3] Lee HY Chen PS Wu TY Chen YS Wang CC Tzeng PJ Lin CH Chen F Lien CH Tsai MJ Low power and high speed bipolar switching with a thin reactive Ti buffer layer in robust HfO_2 _based RRAM IEDM Tech Dig 2008 1

[B4] Zhang T Zhang X Ding L Zhang W Study on resistance switching properties of Na_0.5_Bi_0.5_TiO_3 _thin films using impedance spectroscopy Nanoscale Res Lett 2009 4 1309 1314 10.1007/s11671-009-9397-4 20628453 PMC2893894

[B5] Li Y Long S Zhang M Liu Q Shao L Zhang S Wang Y Zuo Q Liu S Liu M Resistive switching properties of Au/ZrO_2_/Ag structure for low-voltage nonvolatile memory applications IEEE Electron Device Lett 2010 31 117 119

[B6] Lee DY Wang SY Tseng TY Ti-induced recovery phenomenon of resistive switching in ZrO_2 _thin films J Electrochem Soc 2010 157 G166 G169 10.1149/1.3428462

[B7] Lin CC Chang YP Ho CC Shen YS Chiou BS Effect of top electrode materials on the nonvolatile resistive switching characteristics of CCTO films IEEE Trans Magn 2011 47 633 636

[B8] Sun X Li G Chen L Shi Z Zhang W Bipolar resistance switching characteristics with opposite polarity of Au/SrTiO_3_/Ti memory cells Nanoscale Res Lett 2011 6 599 10.1186/1556-276X-6-599 22107926 PMC3260421

[B9] Lin CY Wu CY Wu CY Lee TC Yang FL Hu C Tseng TY Effect of top electrode material on resistive switching properties of ZrO_2 _film memory devices IEEE Electron Device Lett 2007 28 366 368

[B10] Wu X Zhou P Li J Chen LY Lv HB Lin YY Tang TA Reproducible unipolar resistance switching in stoichiometric ZrO_2 _films Appl Phys Lett 2007 90 183507 10.1063/1.2734900

[B11] Lin CY Wu CY Wu CY Lin CC Tseng TY Memory effect of RF sputtered ZrO_2 _thin films Thin Solid Films 2007 516 444 448 10.1016/j.tsf.2007.07.140

[B12] Lin CY Wang SY Lee DY Tseng TY Electrical properties and fatigue behaviors of ZrO_2 _resistive switching thin films J Electrochem Soc 2008 155 H615 H619 10.1149/1.2946430

[B13] Zhou P Shen H Li J Chen LY Gao C Lin Y Tang TA Resistance switching study of stoichiometric ZrO_2 _films for non-volatile memory application Thin Solid Films 2010 518 5652 5655 10.1016/j.tsf.2009.10.034

[B14] Sarma DD Rao CNR XPES studies of oxides of second- and third-row transition metals including rare earths J Electron Spectrosc Relat Phenom 1980 20 25 45 10.1016/0368-2048(80)85003-1

[B15] Colon JL Thakur DS Yang CY Clearfiled A Martin CR X-ray photoelectron spectroscopy and catalytic activity of α-zirconium phosphate and zirconium phosphate sulfophenylphosphonate J Catal 1990 124 148 159 10.1016/0021-9517(90)90111-V

[B16] Kaufmann R Klewe-Nebenius H Moers H Pfennig G Jenett H Ache HJ XPS studies of the thermal behaviour of passivated zircaloy-4 surfaces Surf Interface Anal 1988 11 502 509 10.1002/sia.740111003

[B17] Sun B Liu YX Liu LF Xu N Wang Y Liu XY Han RQ Kang JF Highly uniform resistive switching characteristics of TiN/ZrO_2_/Pt memory devices J Appl Phys 2009 105 061630 10.1063/1.3055414

[B18] Shen W Dittmann R Waser R Reversible alternation between bipolar and unipolar resistive switching in polycrystalline barium strontium titanate thin films J Appl Phys 2010 107 094506 10.1063/1.3369285

[B19] Kwon DH Kim KM Jang JH Jeon JM Lee MH Kim GH Li XS Park GS Lee B Han S Kim M Hwang CS Atomic structure of conducting nanofilaments in TiO_2 _resistive switching memory Nat Nanotechnol 2010 5 148 153 10.1038/nnano.2009.456 20081847

